# Association Between Sleep and Functional Outcome in Critically Ill Patients

**DOI:** 10.21203/rs.3.rs-6977598/v1

**Published:** 2025-07-14

**Authors:** Rebecca Dutta, Leslie C West, Ajay Sampat, Machelle Wilson, Guillermo Palchik, Alan H Yee

**Affiliations:** University of California Davis; UCSF Medical Center; UC Davis: University of California Davis; UC Davis Medical Center; CPMC: California Pacific Medical Center; UC Davis Medical Center

**Keywords:** sleep stages, critically ill, outcome assessment, electrophysiology

## Abstract

**Objective:**

Examine the association between sleep and clinical outcome in patients with acute brain injury and critical illness.

**Methods:**

Retrospective analysis of critically ill patients who underwent continuous electroencephalography monitoring in an academic medical center from 2018–2020. Patients admitted with primary neurologic, medical, and surgical conditions were included. Clinical outcome was determined by the modified Rankin Scale (mRS < 3 represented favorable outcome). Statistical modeling of outcome included predictor variables controlling for anesthetic concentration, diagnosis, and sex.

**Results:**

262 patients were included of which 57% were male with a mean age of 58 years (range 18–91). Twenty-one percent of the total population achieved sleep (56/262). Of those achieving any sleep, 43% had good outcomes compared to only 26% who did not (χ^2^ =10.99, p = 0.0009), controlling for diagnosis, sex, anesthetic level, and Acute Physiology and Chronic Health Evaluation score. Neurological patients attained sleep more often (27%) compared to those with other primary diagnoses (14%). In multivariable analysis, the effect of level of centrally acting anesthetics did not account for sleep differences between neurologic and non-neurological patients (χ^2^ =3.5, p = 0.95).

**Conclusions:**

Neurocritical patients slept more often, and obtaining any sleep was associated with better functional outcome when controlling disease severity. Further studies are needed to determine whether sleep augmentation and anesthetic use in critically ill patients impact functional outcomes.

## Introduction

Sleep is a fundamental dynamic physiologic process essential for cognition, repair, and survival. Disruption of physiologic sleep may have significant implications in acute brain injury, functional recovery, and critical illness. Little is known about the impact of sleep during acute phases of critical illness and its relationship with outcome. Extrapolated data from healthy individuals on how sleep deprivation influences organ function exists; however, limited relevant studies have examined those who are critically ill.^1^

Characterization of sleep in limited medical and surgical intensive care unit (ICU) populations has primarily focused on subjective measurement tools and polysomnography (PSG).^2–5^ Measuring sleep in the ICU is inherently challenging and not synonymous with studying sleep physiology in a standardized laboratory setting. Polysomnography has not been validated for routine use in the critical care setting, but published 24-hour monitoring studies have supported our current understanding of sleep in the ICU.^4^ Sleep fragmentation and frequent arousals are common and often attributed to intrinsic factors to being critically ill, mechanical ventilation, along with extrinsic factors (i.e., care interruptions, light, disruptive noise).^3^ Short sleep periods with unusual sleep stage transitions have been observed along with substantial sleep fragmentation. In an observational ICU cohort study, patients slept only three minutes before waking based on PSG.^5^ Moreover, critically ill patients have substantial circadian misalignment, sleeping primarily during daytime hours.^2,5^ They have markedly abnormal sleep architecture predominantly composed of non-rapid eye movement (REM) light sleep stages (i.e. N1 and N2), reduced N3 slow wave stage, and nearly absent REM.^2–3,5^

Limited evidence exists examining sleep in neurocritical patients.^6^ In a small sample of patients with aneurysmal subarachnoid hemorrhage (SAH) that focused on an isolated sleep stage, 85% did not achieve normal N2 stage within 24 hours of admission while 77% did not sleep at all.^7^ The absence of electroencephalographic (EEG) identification of any sleep architecture was associated with poor 3-month outcome.^7^ Herein, we report the largest study of mixed-population (neurological, medical, surgical) critically ill patients examining electrophysiological sleep characteristics and their association with clinical outcomes.

## Methods

We performed a retrospective analysis of consecutive critically ill patients who underwent continuous EEG (cEEG) monitoring at an academic medical center from January 2018 to December 2020. Adult patients aged 18 years or greater admitted to an ICU with an acute neurological, medical, or surgical condition were included. All underwent cEEG monitoring for at least 24 hours but no more than 7 days as part of routine care. Example indications for monitoring included evaluation for subclinical seizures, encephalopathy, or detection of delayed cerebral ischemia in patients with SAH. Electroencephalographic studies were independently reviewed by board-certified clinical neurophysiologists/epileptologists. Sleep was defined by American Academy of Sleep Medicine (AASM) electrophysiological criteria and documented if a patient achieved sleep for at least one hour throughout the continuous recording. Stage of sleep was defined as non-REM (NREM) stage N1, stage N2, stage N3 and Stage REM in accordance with standard EEG criteria. Primary admission diagnosis, Acute Physiology and Chronic Health Evaluation II (APACHE II) score on admission (Table 1), demographics, relevant medications (e.g., anesthetic/sedation), and EEG characteristics were collected. Centrally acting anesthetic-sedation of propofol or midazolam were collected and tiered in three categories based on dose (Table 2). If combined sedation was used, the patient was tiered into the appropriate medium or high level based on dose.

The functional global outcome was assessed by hospital discharge modifi ed Rankin Scale score (mRS;range: 0 = no symptoms, 6 = death) and dichotomized by good outcome (mRS < 3) versus poor (3–6). Ascore of less than three represented patients with no to minimal neurologic disability.^8^

### Statistical analysis

Statistical analysis was performed to evaluate the effect of sleep on good outcome (mRS < 3). Differences between categorical variables were assessed using chi-square and between continuous variables using *t* tests or Wilcoxon rank sum tests where appropriate. Subsequent multivariable analysis was performed to evaluate the influence of sleep on outcome, accounting for level of anesthetic, and controlling for APACHE II scores, diagnosis, and sex. Sedation intensity was grouped into two categories based on average dose while undergoing cEEG monitoring (none/low level versus moderate/high levels) (Table 2). ^9^ All analyses were conducted using SAS^®^ software version 9.4 for Windows^®^ (SAS Institute Inc., Cary, NC).

### Standard Protocol Approvals, Registrations, and Patient Consents

The University of California Davis Institutional Review Board reviewed and approved the study. The ethics board determined that participant consent was not required. All data were compiled in a secure customized Research Electronic Data Capture (REDCap) database.

## Results

### Patient Characteristics

Two hundred sixty-two patients were included with a mean age of 58 years (range 18–91), and 57% were male. Sixty percent were admitted for a primary neurocritical or neurosurgical diagnosis whereas 40% of patients had medical or surgical critical conditions. Most patients (94%) received mechanical ventilation while undergoing cEEG monitoring with an average recording period of 53 hours. Primary neurological diagnoses included traumatic brain injury [TBI; traumatic subarachnoid hemorrhage (tSAH), subdural hemorrhage (SDH)], acute stroke [ischemic, spontaneous intracerebral hemorrhage (ICH), aneurysmal subarachnoid hemorrhage aSAH)], and status epilepticus. Most primary non-neurologic critical illness diagnoses included respiratory failure, metabolic, toxic or infectious disturbance, sepsis, and cardiac arrest. Additional neurologic and non-neurological diagnoses are itemized in Table 3.

### Patient Outcomes

**Figure 1:**
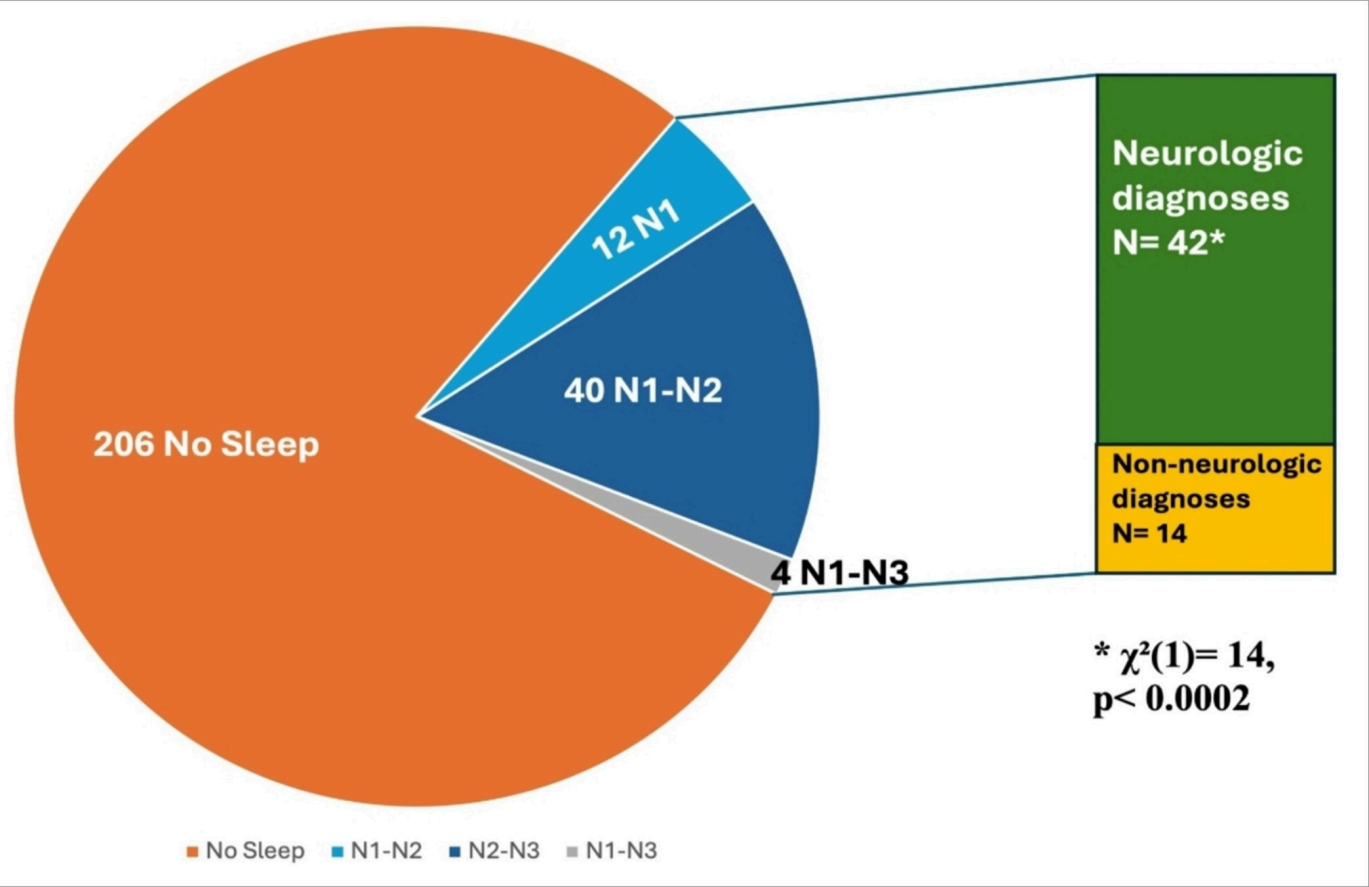
Sleep Stage. Sleep stages for all ICU patients: 56 patients achieved electrophysiologic sleep of which 75% were diagnosed with a primary neurological condition.

Twenty-one percent of all patients (56/262) achieved any sleep. Of those who slept, 21% achieved N1 only, 71% achieved N1 with N2, and only 7% achieved all 3 non-REM stages (N1 to N3). Neurologic patients attained sleep significantly more frequently compared to those with other primary diagnoses (Fig. 1). Thirty percent of all patients died during hospitalization.

Those who achieved any sleep were more likely to have favorable functional outcomes compared to those who did not (p < 0.0004), as well as male sex (P < 0.0074) and non-neurologic diagnosis (P < 0.0011) in univariate analysis. When controlling for diagnosis, sex, anesthetic, and APACHE score [OR = 3.56, 95% CI (1.68, 7.58)], patients who slept in the ICU were 3.6 times more likely to have a good outcome. Furthermore, more favorable outcomes were predicted by those admitted with non-neurological diagnoses (OR = 4.21, 95% CI = (2.17,8.18)) and male sex [OR = 2.24, 95% CI(1.17,4.30)]. The APACHE score, however, did not influence outcome in multivariate analysis (χ^2^ (1)=, 3.48, p = 0.062). Critical ill patients achieved sleep more often in the absence of centrally-acting (e.g. propofol, midazolam) anesthetic use (27.4%) and low level of sedation (26.7%). In contrast, only 6.7% of patients receiving moderate to high anesthetic levels had documented sleep. However, the intensity of anesthetic use did not impact outcome (χ^2^(2) = 0.11, P < 0.95).

## Discussion

Little is known about the impact of sleep on clinical outcomes in survivors of critical illness, particularly in those with severe brain injury. We examined a broad spectrum of critical patients and identified key characteristics associated with sleep and functional outcomes. Very ill patients seldom sleep and when they do, achieve incomplete restful sleep (only lighter stages of NREM sleep, stages N1-N2). Importantly, attaining any sleep was associated with better functional outcomes.

Surprisingly, sleep was identified more commonly in patients with primary brain injury compared to those admitted for other conditions. The difference in achieving sleep between those with neurologic or non-neurological disease did not result from sedative use. Sleep disturbances (primarily hypersomnia) can be seen from disturbance in the wakeful regions of the brain (i.e. reticular activating system, hypothalamus, areas surrounding third ventricle, thalamus) or due to hormonal imbalance (i.e. reduction in histamine and hypocretin concentrations, wake-promoting neurochemicals, are observed following TBI) which may explain some hypersomnia features.^10,11^ We hypothesized that neurologically injured patients would achieve less sleep based on involvement of key anatomical structures.^12^ However, those admitted for medical critical illness slept less often than those with neurocritical disease in our analysis. Diffuse systemic inflammatory processes, such as sepsis, may affect sleep more severely by disrupting blood-brain barrier integrity.^13^ Furthermore, critical systemic illness may disrupt the normal circadian rhythm.^14,15^

Patients who slept less in our cohort had poorer functional outcomes. Short and long-term effects of sleep loss may impair immune recovery while negatively impacting cardiovascular and pulmonary function.^1^ Polysomnography (PSG) is a reliable outpatient method used to qualify, and quantify, sleep. However, PSG has not been validated to examine sleep in the ICU. Continuous electroencephalographic monitoring is increasingly used in the critically-ill and may serve as a useful surrogate biomarker for sleep characterization. Actigraphy, a non-invasive worn device measuring rest and activity cycles, has tested circadian rhythm timing and sleep-associated variables in the critical care setting.^17–21^ However, this modality does not record cerebral electrophysiology, thus conclusions about sleep can only be extrapolated. Improved identification of sleep-associated variables, based on AASM standardized metrics, can be better characterized (i.e. sleep stages) by cEEG monitoring.^22^

Recent critical care guidelines proposed early progressive mobilization to prevent delirium; importantly, the report underscored sleep promotion and minimizing sleep disruption as key strategies to impact survivor outcome.^21^ Patients with prior brain injury are at risk of developing circadian rhythm dysfunction. Stroke and TBI survivors are 40% more likely to be diagnosed with a sleep disorder, and little is known about predisposing risks or impact of interventions to minimize sleep disruption in this population.^23,24^ Other important variables that may influence sleep among the critically ill, such as the potential impact of continuous enteral feeding, require ongoing investigation.^25,26^

We examined a diverse population of critically ill patients and evaluated the effect of commonly used sedatives on sleep. Expectedly, patients achieved sleep more often when these medications were not used; however, anesthetic administration did not exclusively predict why those with neurological injury achieved sleep more often compared to those without brain insults.

### Limitations

Inherent limitations exist with any single center, retrospective study. However, uniformity in how clinical practice is administered and information collected should minimize data variability. Several useful clinical variables were not systematically recorded such as the Glasgow Coma Scale in medical and surgical patients, individual circadian sleep timing, or pre-existing sleep disorder diagnoses due to low prevalence. Study patients underwent cEEG monitoring for various clinical indications, generating a potential bias towards those with greater medical complexity who may have less “central neurologic reserve.”

Examining how these variables as well as other pharmacologic therapies (i.e. anticonvulsants, antipsychotics, antimicrobials, opiates, tricyclics and other antidepressants) might influence sleep in future studies will be informative. Lastly, dexmedetomidine was an anesthetic infrequently used in our cohort and not analyzed independently—its application in delirium prevention in critically ill patients is becoming increasingly recognized.^27^

## Conclusion

Most critically ill patients do not achieve electrophysiologic sleep and those that do reach N1/N2 stages only. However, achieving any sleep was associated with more favorable functional outcome, and neurological patients slept more often. Sleep appears to be an important variable in the critically ill even when reaching ‘lighter’ restorative stages. Further studies are needed to standardize measurements of sleep in the ICU, determine effective interventions that promote it, and examine the impact of sleep on critical care outcomes.

## Supplementary Material

Supplementary Files

This is a list of supplementary files associated with this preprint. Click to download.

• Table3.docx

• SupplementaryMaterial.docx

## Figures and Tables

**Table 1:** APACHE II Score APACHE II, Acute Physiology and Chronic Health Evaluation II.

Diagnosis	APACHE II Score	
Neurologic & Neurosurgical Diagnosis	Average	**14.97**
	Standard Deviation	6.40
Medical & Surgical Diagnosis	Average	**18.12**
	Standard Deviation	7.82
Total Average of APACHE II Score		**16.22**
Total Standard Dev of APACHE II Score		**7.15**

Acute Physiology and Chronic Health Evaluation, APACHE

**Table 2:** Central Acting Anesthesia Dose by Dosage Tiers Microgram, mcg; kilogram, kg

Tier	Sedative Regimen
None	None
Low dose	Propofol < 20 mcg/kg/minute OR Midazolam < 2 mcg/kg/minute
Moderate-to-high dose	Combination of propofol and midazolam at any dose OR Propofol ≥ 20 mcg/kg/minute OR Midazolam ≥ 2 mcg/kg/minute

Microgram, mcg; kilogram, kg
